# Analyzing the efficiency of small and medium-sized enterprises of a national technology innovation research and development program

**DOI:** 10.1186/2193-1801-3-403

**Published:** 2014-08-04

**Authors:** Sungmin Park

**Affiliations:** Department of Business Administration, Baekseok University, Cheonan, ROK 330-704 Korea

**Keywords:** Data envelopment analysis, Diseconomies of scale, Efficiency, Joint venture, Kruskal-Wallis test, R&D performance evaluation, Small and medium-sized enterprise

## Abstract

This study analyzes the efficiency of small and medium-sized enterprises (SMEs) of a national technology innovation research and development (R&D) program. In particular, an empirical analysis is presented that aims to answer the following question: “Is there a difference in the efficiency between R&D collaboration types and between government R&D subsidy sizes?” Methodologically, the efficiency of a government-sponsored R&D project (i.e., GSP) is measured by Data Envelopment Analysis (DEA), and a nonparametric analysis of variance method, the Kruskal-Wallis (KW) test is adopted to see if the efficiency differences between R&D collaboration types and between government R&D subsidy sizes are statistically significant. This study’s major findings are as follows. First, contrary to our hypothesis, when we controlled the influence of government R&D subsidy size, there was no statistically significant difference in the efficiency between R&D collaboration types. However, the R&D collaboration type, “SME-University-Laboratory” Joint-Venture was superior to the others, achieving the largest median and the smallest interquartile range of DEA efficiency scores. Second, the differences in the efficiency were statistically significant between government R&D subsidy sizes, and the phenomenon of diseconomies of scale was identified on the whole. As the government R&D subsidy size increases, the central measures of DEA efficiency scores were reduced, but the dispersion measures rather tended to get larger.

## Introduction

Based on a program logic model, public sector research and development (R&D) performance can be evaluated, and the performance efficiency and effectiveness of government-sponsored R&D projects (i.e., GSPs) is analyzed using a variety of methodologies (Wholey 1983; Bickman 1987; Wholey 1987). The W. K. Kellogg Foundation (WK Kellogg Foundation (WKKF) (2004)) classified performance as follows according to three different time periods during which it occurs: (1) short-term (1 ~ 3 years) output, (2) mid-term (4 ~ 6 years) outcome, and (3) socioeconomic long-term (7 ~ 10 years) impacts, most of which occur after related activities are completed. Some typical flow-chart type program logic models in the literature are the Advanced Technology Program (ATP) logic model of U.S. Department of Commerce (DOC) (Ruegg and Feller 2003) and the Research and Technology Development and Deployment Program (RTDDP) logic model of U.S. Department of Energy (DOE) (McLaughlin and Jordan 1999). Stainer and Nixon (1997) stressed that R&D performance evaluation might be difficult, especially due to the nature of the long-term performance, but it should be vital to the success of R&D strategic planning.

Representative factors for public sector R&D inputs and the three-phased subsequent performance are summarized as follows. For the R&D inputs, there are budget, staff members, and so on. Meanwhile, the intellectual property-related output includes publications, patents, and the commercialization-related outcome includes new or improved products, processes and services, and firm growth. For the socioeconomic long-term impacts, employment gains and international competitiveness are considered (McLaughlin and Jordan 1999; Ruegg and Feller 2003; WK Kellogg Foundation WKKF 2004). Specifically, Hsu and Hsueh (2009) presented a summary of representative factors of the R&D inputs and performance for the efficiency analysis of a government-sponsored R&D project. For R&D inputs, four factors were listed: a government R&D subsidy to GSP, GSP budget from government subsidy recipient, staff members and the post-project period. The four performance factors were published articles, patent applications and registrations, patents used and profited commercialization.

As described in Section 2 of the background for this study in detail, in the recent deployment of some national R&D programs, the importance of interagency R&D collaboration has been underlined. In addition, it is verified that the government R&D subsidy to GSP has been supervised within an appropriate size limit.

However, in the case of national technology innovation, in R&D programs especially, previous studies are very limited so far regarding whether R&D collaboration as well as government R&D subsidies really matter in creating performance. Also, one of the common limitations of related studies analyzing R&D productivity is not to reflect sufficiently the time-lag between the R&D inputs and performance (Wu et al. 2006; Guan and Chen 2010; Chen et al. 2011). In addition, proxy variables used for representing the R&D productivity and the level of technology innovation were somewhat conceptual. From a methodological perspective, it can be pointed out that most of related studies mainly relied on regression models using survey data (Fritsch and Lukas 2001; Belderbos et al. 2004; Laursen and Salter 2006; Berchicci 2013; Robin and Schubert 2013). Meanwhile, it should be noted that R&D performance evaluation may not be easy in the real world, especially associated with technology innovation R&D programs. Due to some inherent reasons (e.g., scarcity of GSPs creating performance), it is difficult to obtain a large enough sample to analyze in the case of technology innovation R&D programs (Shipp et al. 2005; Ruegg 2006; Ministry of Knowledge Economy MKE 2012a).

The present study conducts an empirical analysis in order to answer the following question: “Is there a difference in the efficiency between R&D collaboration types and between government R&D subsidy sizes?” Data Envelopment Analysis (DEA) measures the efficiency of GSP, and a nonparametric analysis of variance method, the Kruskal-Wallis (KW) test, is adopted to determine if the efficiency difference is statistically significant between R&D collaboration types and between government R&D subsidy sizes. The sample to be analyzed in the present study is a set of GSPs carried out by small and medium-sized enterprises (SMEs) among Knowledge Economy Technology Innovation Program (KETIP) of Ministry of Knowledge Economy (MKE) in the Korean government, which is the biggest set of GSPs classified by the recipient types of government R&D subsidy. In particular, KETIP is a representative national technology innovation R&D program in the Korean government that specifically aims to achieve economic sales as well as technical intellectual property. In this context, the majority of the government R&D subsidy recipients consist of SMEs and large companies in KETIP. However, it should be noted that the generic goal of an R&D subsidy policy is to promote R&D investments on to R&D projects where the ratio of social benefits to economic outcomes is high or on to R&D projects which waive strong forms of intellectual property protection.

The main contribution of the present study to the literature is to provide a quantitative nonparametric discussion regarding the R&D efficiency comparisons through an empirical analysis with a massive dataset coping with the time-lag problem properly, which fills the gap in the related literature. The present study is organized as follows: Section 2 states the background of the present study which includes the literature review and the research hypotheses, Section 3 explains the methods and research models and Section 4 presents the results and discussion associated with the design of the sample as well as the empirical analysis. Finally, conclusions are summarized in Section 5. Hereafter, unless stated otherwise, the term “collaboration type” refers to the R&D collaboration type, and the term “government subsidy” refers to the government R&D subsidy.

## Background

DEA is a common methodology for evaluating R&D performance efficiency (Rouse and Putterill 2003; Bitman and Sharif 2008). Lee et al. (2009) used a DEA/AR model in their R&D performance efficiency study, with six heterogeneous public R&D programs. Sharma and Thomas (2008) compared the national R&D programs of 22 countries, and they found that a small number of R&D programs carried out by developing countries were benchmarks located on the efficiency frontier.

Chen et al. (2011) compared the productivity change of 73 information technology (IT) companies in China using a DEA-based Malmquist Index (MI) model. They argued that R&D collaboration was necessary between large companies and SMEs. Belderbos et al. (2004) analyzed the influence of R&D collaboration on the labor productivity and the technology innovation productivity of 2,056 companies in the Netherlands for the three years from 1996 to 1998 based on a regression model, and it was found that the productivity pursued by each R&D collaboration type was different from one another regarding four R&D collaboration types. Fritsch and Lukas (2001) examined a survey sample of 1,800 German companies based on a two-stage decision-making model (i.e., 1st stage: a Logit model, 2nd stage: a Poisson regression model), and they verified a statistically significant positive correlation (+) between the R&D collaboration frequency and the firm size (i.e., the number of employees) as well as the R&D intensity (i.e., the percentage of R&D employees).

Berchicci (2013) analyzed a survey sample of 2,537 Italian firms for the 13 years from 1992 to 2004 using a Tobit regression model, and it was argued that an inverted U-shape relationship existed between the share of external R&D activities and the firm’s innovative performance (i.e., the share of turnover from new or significantly improved products). In particular, for the firms with high R&D capacity, the optimal value of external R&D activity share for which the maximum performance value achieved was at 26.7%, while the equilibrium point was at 42% for the firms with low R&D capacity. Robin and Schubert (2013) carried out a survey associated with French and German companies for the five years from 2004 to 2008, and they found that the R&D collaboration increased only the product innovation (i.e., the percentage of total sales due to new products). However, they also required a careful interpretation of their results, because the results were dependent on the countries, industry types and periods analyzed. Laursen and Salter (2006) investigated a survey sample of 2,707 U.K firms in 2001 using a Tobit regression model, and they identified that a dependent variable for the innovative performance (i.e., the fraction of the firm’s turnover relating to products new to the world market) was curvilinearly related to the external knowledge search breadth and depth, taking an inverted U-shape.

From a theoretical perspective, Ruegg and Feller (2003) presented an excellent summary on the R&D collaboration propensity as a key factor for the success of GSPs. Also, Geuna et al. (2003) and Stephan (2010) provided a discussion associated with innovative R&D projects in the light of R&D funding as well as the intangible R&D capabilities affecting the overall R&D productivity. Meantime, David et al. (2000) presented an excellent survey of the econometric literature regarding the relationship between public and private R&D spending. Conceptually, Klette et al. (2000) reviewed some econometric studies evaluating effects of commercial GSPs, and they examined not only where the public R&D expenditure stimulated innovative activities but also to what extent potential R&D spillovers were internalized in the market.

Additionally, García-Quevedo (2004) analyzed the relationship between public funding of R&D and private R&D expenditures. Cerulli (2010) scrutinized principal econometric models used to measure the effects of public support for firm R&D investment. Zúñiga-Vicente et al. (2014) summarized some empirical literature on the relationship between public R&D subsidies and private R&D investment in order to understand potential effects of public R&D subsidies on private R&D spending.

In particular, Esteve-Pérez and Rodríguez (2013) analyzed a sample of Spanish manufacturing SMEs drawn from a Business Strategy Survey over the 1990–2006 period, and they argued that some types of R&D collaboration with suppliers, clients, universities and technological centers could be more relevant for SMEs to access international practices and technology innovation activities. Based on 1,435 SMEs in Australia during the 2004–2007 period, Gronum et al. (2012) examined the role of networks in SMEs, and they showed that SMEs’ strong and heterogeneous ties engaged with different actors improved the innovation and long-term performance in SMEs. Ortega-Argilés et al. (2009) mention that innovative SMEs tend to rely heavily on external knowledge that is a crucial complement to in-house R&D and innovation management practices. Based on the previous studies described above, the first hypothesis of the present study can be stated as follows:

### Hypothesis 1

There is a difference in R&D performance efficiency between GSP’s collaboration types of a national technology innovation R&D program.

Hsu and Hsueh (2009) evaluated DEA efficiency of 110 GSPs, and consequently they emphasized the need for an appropriate upper limit on the ratio of the amount of government support in the GSP’s R&D budget. Tsai (2005) examined a panel sample of 82 high-tech manufacturing enterprises in Taiwan for the six years from 1995 to 2000 using a regression model associated with the Cobb-Douglas production function, and it mentioned that there was a nonlinear U-type relationship between the firm’s total factor productivity (TFP) and its firm size (i.e., the number of employees and the total fixed assets). It implies that both large and small firms have higher competitive advantage in terms of TFP than moderate-sized firms. Regarding pharmaceutical and semiconductor companies, Kim et al. (2009) argued that the number of patents per R&D expenditure declined with firm size (i.e., the firm sales) for both industries based on regression analyses. In summary of the previously aforementioned studies, the second hypothesis of the present study is formulated as follows:

### Hypothesis 2

There is a difference in R&D performance efficiency between GSP’s subsidy sizes of a national technology innovation R&D program.

## Methods and research models

### Efficiency analysis - DEA

DEA is a methodology of Operations Research (OR) that calculates relative efficiency scores for a set of peer entities in the range of [0, 1]. Each entity pursuing the same objective, called a Decision Making Unit (DMU), has common multiple input–output variables. A variety of DEA-related literature has been published since the model from Charnes, Cooper and Rhodes (CCR) was proposed based on the assumption of Constant Returns to Scale (CRS) for the first time (Charnes et al. 1978). Then, the Banker, Charnes and Cooper (BCC) model was established with a Variable Returns to Scale (VRS) assumption. The BCC model can be regarded as a variant of the CCR model (Banker et al. 1984). In the beginning, the active fields of DEA applications were hospitals (Banker et al., 1986), education programs (Charnes and Cooper 1980; Charnes et al. 1981; Bessent et al. 1982), urban police department (Parks 1983), banking centers (Sherman and Gold 1985) and so on. Furthermore, Seiford and Thrall (1990), Callen (1991), Zhu (2003), Cooper et al. (2004) and Cooper et al. (2007) provide excellent explanations for the mathematical formula and computational implementation for various DEA models as follows.

Assume a set of *n* DMUs (*j* = 1, …, *n*) having *m* input variables (*i* = 1, …, *m*) and *s* output variables (*r* = 1, …, *s*). Then, for each DMU_*j*_, semipositive vectors of input–output variables are defined as , . For all DMUs, the matrices of input–output variables are defined as , . Eq.(1) is an input-oriented DEA model that calculates a DEA efficiency score  of DMU_*o*_ (Zhu 2003; Cooper et al. 2004; Cooper et al. 2007). In Eq.(1), every element of  has a value of zero, all elements of  are ones, and a semipositive  is a vector of DMU intensity.
1

The production possibility set *P* enveloped by the frontier is defined as Eq.(2). Here,  corresponds to two different Returns to Scale (RTS) assumptions, such as CRS and VRS, respectively.
2

Referring to the literature relating to national R&D programs including Ruegg and Feller (2003), Hsu and Hsueh (2009), and Guan and Chen (2010), DEA input and output variables in the present study are selected in Table 1. For the input variables, four characteristics are considered, including Government subsidy (*x*_1_), Budget from recipient (*x*_2_), R&D staffs (*x*_3_) and R&D period (*x*_4_). On the other hand, for the output variables, three typical performance factors are selected such as Publications (*y*_1_), Patents (*y*_2_) and Commercialization sales (*y*_3_). Consequently, a total of seven variables comprise the DEA model in the present study to analyze the efficiency of each GSP (i.e., DMU). Regarding the two output variables (i.e., Publications (*y*_1_) and Patents (*y*_2_)), each variable is defined as a weighted sum of its own sub-items in Table 1, and the weights used are obtained from the reference of a KETIP’s performance index design guideline (Ministry of Knowledge Economy MKE 2012b). In the preparation of the sample in Section 4, the exchange rate of 1,000 Won/US$ 1 is applied to the raw data to convert monetary units, and the matching fund, that is Budget from recipient (*x*_2_), corresponds to the amount of cash inflow from the government subsidy recipient exclusive of any nonmonetary investment.

**Table 1:** **DEA input–output variables**

DEA input variable	Variable name	Sub-item	Unit of variable
Government subsidy	*x*_1_		(US$ 10^6^)
Budget from recipient	*x*_2_		(US$ 10^6^)
R&D staffs	*x*_3_		
R&D period	*x*_4_		Years
**DEA output variable**	**Variable name**	**Sub-item**	
Publications	*y*_1_ = 1.0 × *y*_*a*_ + 0.5 × *y*_*b*_	SCI articles (*y*_*a*_)	
		Non-SCI articles (*y*_*b*_)	
Patents	*y*_2_ = 1.0 × *y*_*c*_ + 0.2 × *y*_*d*_ + 0.6 × *y*_*e*_ + 0.2 × *y*_*f*_	Foreign registrations (*y*_*c*_)	
		Foreign applications (*y*_*d*_)	
		Domestic registrations (*y*_*e*_)	
		Domestic applications (*y*_*f*_)	
Commercialization sales	*y*_3_		(US$ 10^6^)

### Nonparametric analysis of variance - KW test

As explained in Section 4.3 and 4.4, it is not valid to test statistical significance of the efficiency difference, assuming a specific probability distribution for the population of DEA efficiency scores. Therefore, a nonparametric (i.e., distribution-free) analysis of variance method, KW test is used to see if the efficiency difference is statistically significant between R&D collaboration types and between government R&D subsidy sizes.

Suppose that  is the total number of observations. Rank all *n* observations from smallest to largest, and assign the smallest observation as rank 1 and the largest observation as rank *n*. When observations are tied, assign an mean rank to each of the tied observations. Eq.(3) is the tied-ranks adjusted KW test statistic, which requires the same continuous probability distribution for each level *i* = 1, …, *a*. In Eq.(3), the notation of Eq.(4) is used, which is just the variance of the ranks (Montgomery 1997; Minitab 2005). In Eq.(3) and Eq.(4),  denotes the total of the  ranks in the *i*th level, and  denotes the rank of observation  (i.e., the *j*th observation within the *i*th level). In fact, Eq.(3) measures the degree to which the actual observation mean ranks  differ from their expected value of  under the null hypothesis , where  is the population median of the *i*th level.
34

In practice, the following large-sample approximation, Eq.(5) is usually employed for determining the minimum sample size. When the size of sample meets the conditions in Eq.(5), the null hypothesis  can be rejected if the observed value of  with *a -* 1 degrees of freedom and the significance level of α.
5

In addition, Eq.(6) (i.e., the standardized Z-value of ) can be very useful for individual comparisons between levels, which indicates how far  differs from  (Minitab 2005).
6

## Results and discussion - an empirical analysis

### Description of the sample

As mentioned earlier, the sample to be analyzed in the present study is a set of GSPs conducted by SMEs among KETIP in the year of 2012, which is the biggest set of GSPs classified by the recipient types of government R&D subsidy. In fact, KETIP 2012 investigated all completed R&D projects for the five performance follow-up survey years from 2007 to 2011. Therefore, this R&D performance investigation can be regarded as a system that fully considers the time-lag between the R&D inputs and performance (Government Performance Results Act GPRA 1993; Ministry of Science Technology MST 2007; Ministry of Knowledge Economy MKE 2012a). The number of GSPs conducted by SME is 1,899 during that period of time. Table 2 summarizes numbers of GSPs and their proportions creating performance regarding three DEA output variables within the sample of 1,899 GSPs. Actually, the data set of the sample was downloaded from the “e-R&D” Database Management System (DBMS) of Korea Evaluation Institute of Industrial Technology (KEIT) under MKE. KEIT has its own Web-based annual survey system where GSP subsidy recipients should enter their R&D performance data, and the reliability of the data entered is fully verified by the National Science and Technology Information Service (NTIS) system administered by Korea Institute of S&T Evaluation and Planning (KISTEP). In obtaining the sample from the “e-R&D” system, the field of “R&D institution type” was filtered in order to extract GSPs carried out by SMEs only. Therefore, the selection criterion for SMEs in this study is identical to the KEIT’s definition.

**Table 2:** **Summary of GSPs associated with the government R&D subsidy recipient type of SME**

DEA output variable	Variable name	Number of GSPs without performance	Number of GSPs with performance	Total	Proportion (%)
			(a)	(b)	(a)/(b)
Publications	*y*_1_	1,424	475	1,899	25.01
Patents	*y*_2_	1,018	881	1,899	46.39
Commercialization sales	*y*_3_	1,155	744	1,899	39.18
	*y*_1_ & *y*_2_		368	1,899	19.38
	*y*_1_ & *y*_3_		217	1,899	11.43
	*y*_2_ & *y*_3_		435	1,899	22.91
	*y*_1_ & *y*_2_ & *y*_3_		178	1,899	9.37

As we can see in Table 2, the number of GSPs with Publications (*y*_1_) is 475 (25.01%), and the number of GSPs with Publications (*y*_1_) as well as Patents (*y*_2_) is 368 (19.38%). Consequently, only 178 (9.37%) GSPs created performance for all three DEA output variables. Hence, it is known to be very hard in reality for a GSP to generate Commercialization sales (*y*_3_), which is one of the typical mid-term and economic outcomes, especially in this kind of national technology innovation R&D program considered.

Among those 178 GSPs, 39 GSPs are excluded from the analysis afterward because these have a value less than US$ 0.01 × 10^6^ in either of the two DEA input variables such as Government subsidy (*x*_1_) and Budget from recipient (*x*_2_). Consequently, a sample of *n*_1_ = 139 GSPs is prepared for the efficiency comparisons. Table 3 shows the composition of the sample according to the government R&D subsidy size per GSP as well as the R&D collaboration type in the present study.

**Table 3:** **Summary of the sample (i.e., GSPs analyzed)**

Government subsidy per GSP	GS-Class	^(1)^JV-Type1	^(2)^JV-Type2	^(3)^JV-Type3	Single-company	Total	Proportion (%)
≤ US$ 1 × 10^6^	GS-Class1	12	14	2	6	34	24.46
(US$ 1 × 10^6^, US$ 2 × 10^6^]	GS-Class2	19	24	20	6	69	49.64
(US$ 2 × 10^6^, US$ 3 × 10^6^]	GS-Class3	5	5	8	2	20	14.39
(US$ 3 × 10^6^, US$ 4 × 10^6^]	GS-Class4	2	3	2	1	8	5.76
US$ 4 × 10^6^ <	GS-Class5		1	7		8	5.76
Total		38	47	39	15	139	100.00

^(1)^JV-Type1: SME-Laboratory Joint-Venture.

^(2)^JV-Type2: SME-University Joint-Venture.

^(3)^JV-Type3: SME-University-Laboratory Joint-Venture.

The collaboration type is separated into four different categories. JV-Type1 indicates SME-Laboratory Joint-Venture, JV-Type2 indicates SME-University Joint-Venture, and JV-Type3 denotes the Joint-Venture composed of three distinctive institutions including SME-University-Laboratory. The last one, Single-company, means that an SME conducts its GSP solely without any R&D collaboration with the other types of institution. In terms of the collaboration type, the number of observations within JV-Type2 47 (33.81%) is the largest. From the government subsidy size perspective, the largest number of observations, 69 (49.64%) GSPs belong to GS-Class2, in which the government subsidy per GSP locates in the range of (US$ 1 × 10^6^, US$ 2 × 10^6^]. Table 4 displays descriptive statistics regarding seven DEA input and output variables associated with the sample of *n*_1_ = 139.

**Table 4:** **Descriptive statistics of the sample (** ***n***_**1**_ **= 139)**

	Government subsidy	Budget from recipient	R&D staffs	R&D period	Publications	Patents	Commercialization sales
	*x*_1_	*x*_2_	*x*_3_	*x*_4_	*y*_1_	*y*_2_	*y*_3_
Min	0.11	0.01	1.00	1.00	0.25	0.20	0.01
Max	6.64	1.66	73.00	9.75	18.00	56.80	52.28
Median	1.51	0.29	16.00	3.00	1.50	1.60	0.80
Mean	1.76	0.41	18.78	3.49	2.90	3.45	4.46
IQR	1.02	0.46	14.00	2.83	2.00	3.40	4.86
StDev	1.17	0.36	12.61	1.70	3.43	5.85	8.39
Sum	244.21	56.51	2,610.00	484.58	403.00	480.12	620.37

### Correlation analysis between R&D inputs and performance

Table 5 shows Pearson’s *r* correlation coefficients of the sample of *n*_1_ = 139 between DEA input and output variables. In particular, as discussed briefly in Section 4.1, *r* = 0.118 (P-value = 0.165) between Government subsidy (*x*_1_) and Commercialization sales (*y*_3_) has a positive (+) sign, but there is no statistical significance at all. On the other hand, Publications (*y*_1_) as well as Patents (*y*_2_) are short-term and technical outputs that have a statistically significant positive (+) correlation with Government subsidy (*x*_1_). Therefore, the impact of government support on creating R&D performance can be limited within the range of short-term and technical outputs.

**Table 5:** **Pearson’s** ***r*** **correlation coefficients of the sample (** ***n***_**1**_ **= 139)**

			Government subsidy	Budget from recipient	R&D staffs	R&D period	Publications	Patents
			*x*_1_	*x*_2_	*x*_3_	*x*_4_	*y*_1_	*y*_2_
Budget from recipient	*x*_2_	(*r*)	0.657^***^					
		(P-value)	(0.000)					
R&D staffs	*x*_3_		0.533^***^	0.318^***^				
			(0.000)	(0.000)				
R&D period	*x*_4_		0.469^***^	0.491^***^	0.046			
			(0.000)	(0.000)	(0.588)			
Publications	*y*_1_		0.383^***^	0.233^***^	0.185^**^	0.360^***^		
			(0.000)	(0.006)	(0.029)	(0.000)		
Patents	*y*_2_		0.199^**^	0.261^***^	0.163^*^	0.125	0.325^***^	
			(0.019)	(0.002)	(0.056)	(0.143)	(0.000)	
Commercialization sales	*y*_3_		0.118	0.260^***^	0.195^**^	0.119	0.188^**^	0.282^***^
			(0.165)	(0.002)	(0.022)	(0.163)	(0.027)	(0.001)

^*^, ^**^, ^***^indicate statistical significance at the significance level α = 10%, 5%, 1% respectively.

### Comparisons of the efficiency between GSP’s collaboration types

First of all, in order to compare the efficiency of four different collaboration types, there is a need to control the influence of government subsidy size. Therefore, it is desirable to compare the efficiency independently according to the five different government subsidy sizes shown in Table 3. However, among the sample of *n*_1_ = 139, only the case of GS-Class2 meets the minimum sample size for KW test ().

**Figure 1:**
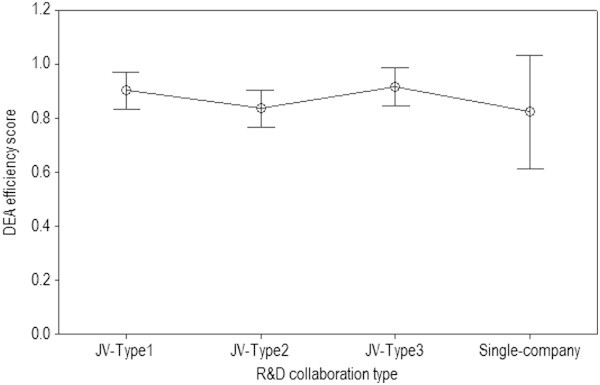
**R&D collaboration type comparisons with the 95% CIs of DEA efficiency scores (** ***n***_**2**_ **= 69).**

Table 6 presents a summary of descriptive statistics of VRS DEA efficiency scores using the sample of *n*_2_ = 69 belonging to GS-Class2 only (DEA-Solver-Pro 2012). Figure 1 shows 95% confidence interval (CI) of DEA efficiency scores for each collaboration type. In terms of the mean of DEA efficiency scores, JV-Type3 has the largest, while Single-company has the smallest. From a dispersion perspective, Single-company can be regard as the worst due to the two largest dispersion measures such as interquartile range (IQR) = 0.386 and standard deviation (StDev) = 0.202.

**Table 6:** **Descriptive statistics of DEA efficiency scores (** ***n***_**2**_ **= 69)**

	JV-Type1	JV-Type2	JV-Type3	Single-company
*n*	19	24	20	6
Min	0.638	0.516	0.560	0.566
Max	1.000	1.000	1.000	1.000
Median	1.000	0.848	1.000	0.871
Mean	0.902	0.836	0.915	0.823
IQR	0.286	0.319	0.217	0.386
StDev	0.142	0.162	0.152	0.202

**Figure 2:**
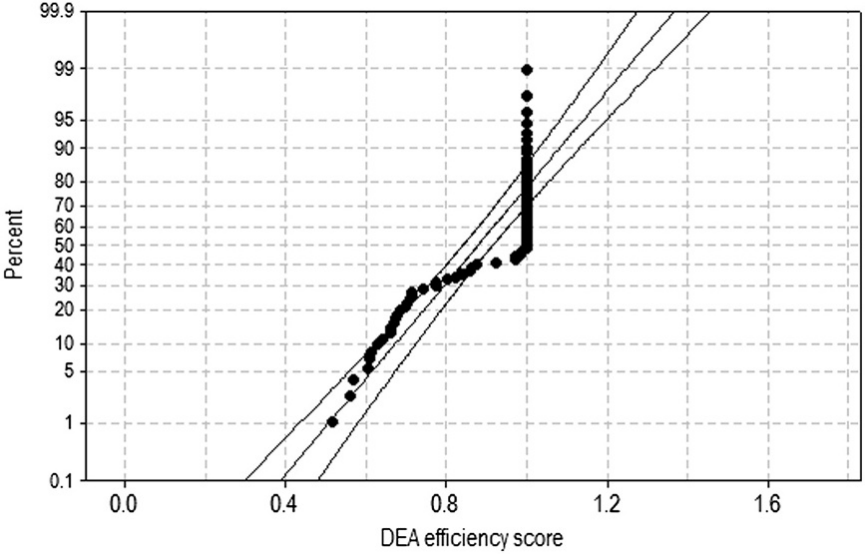
**Normal probability plot of DEA efficiency scores with the 95% CI (** ***n***_**2**_ **= 69).**

Meanwhile, based on the Normal probability plot of DEA efficiency scores with the sample of *n*_2_ = 69 in Figure 2, it is not appropriate to assume the Normality. Additionally, two test statistics such as Anderson-Darling test statistic AD = 7.633^***^ (P-value < 0.005) and Kolmogorov-Smirnov test statistic KS = 0.302^***^ (P-value < 0.010) strongly support that their null hypotheses, assuming the Normality should be rejected. Hereafter, ^*^, ^**^, ^***^ indicate statistical significance at the significance level of α = 10%, 5%, 1%, respectively.

Therefore, it is necessary to compare the efficiency between collaboration types based on a nonparametric analysis of variance method. Table 7 shows KW test results on DEA efficiency scores with the sample of *n*_2_ = 69 in Figure 2. Based on the tied-ranks adjusted test statistic H = 4.12 (P-value = 0.249), the efficiency difference between collaboration types is not statistically significant. Therefore, Hypothesis 1 in Section 2 can be partially accepted because JV-Type3 has the largest median and the smallest dispersion of DEA efficiency scores. On the other hand, Single-company is the worst regarding the two largest dispersion measures.

**Table 7:** **Kruskal-Wallis tests on DEA efficiency scores (** ***n***_**2**_ **= 69)**

***i***	JV-Type	***n***_***i***_	Median	Rank mean	Z _***i***_
1	JV-Type1	19	1.000	36.8	0.46
2	JV-Type2	24	0.848	29.9	-1.53
3	JV-Type3	20	1.000	40.7	1.49
4	Single-company	6	0.871	30.8	-0.54
	Total	69		35.0	

H = 4.12, DF = 3, P-value = 0.249 (tied-ranks adjusted).

### Comparisons of the efficiency between GSP’s subsidy sizes

**Figure 3:**
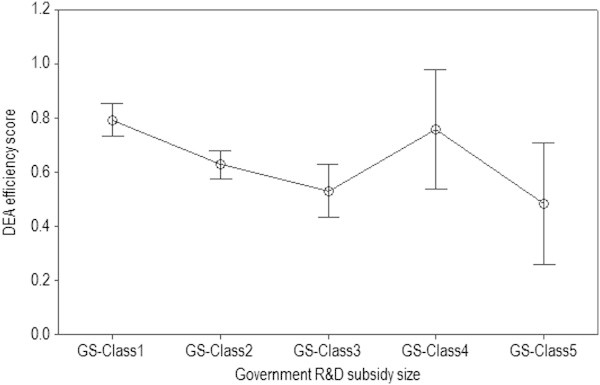
**Government R&D subsidy size comparisons with the 95% CIs of DEA efficiency scores (** ***n***_**1**_ **= 139).**

Because it is determined that the efficiency difference is not statistically significant between R&D collaboration types in Section 4.3, the sample of *n*_1_ = 139 can be pooled and analyzed in order to compare the efficiency between government subsidy sizes. Table 8 presents a summary of descriptive statistics of VRS DEA efficiency scores using the sample of *n*_1_ = 139. Figure 3 shows 95% CI of DEA efficiency scores for each government subsidy size. In terms of the mean of DEA efficiency scores, GS-Class1 has the largest, while GS-Class5 has the smallest. As for the dispersion, the last two sizes, such as GS-Class4 and GS-Class5, are the worst due to the largest dispersion measures. GS-Class4 has IQR = 0.512 and StDev = 0.264, and GS-Class5 has IQR = 0.429 and StDev = 0.271.

**Table 8:** **Descriptive statistics of DEA efficiency scores (** ***n***_**1**_ **= 139)**

	GS-Class1	GS-Class2	GS-Class3	GS-Class4	GS-Class5
*n*	34	69	20	8	8
Min	0.397	0.267	0.261	0.444	0.219
Max	1.000	1.000	1.000	1.000	1.000
Median	0.816	0.588	0.453	0.794	0.436
Mean	0.793	0.628	0.530	0.757	0.483
IQR	0.372	0.247	0.293	0.512	0.429
StDev	0.176	0.216	0.211	0.264	0.271

**Figure 4:**
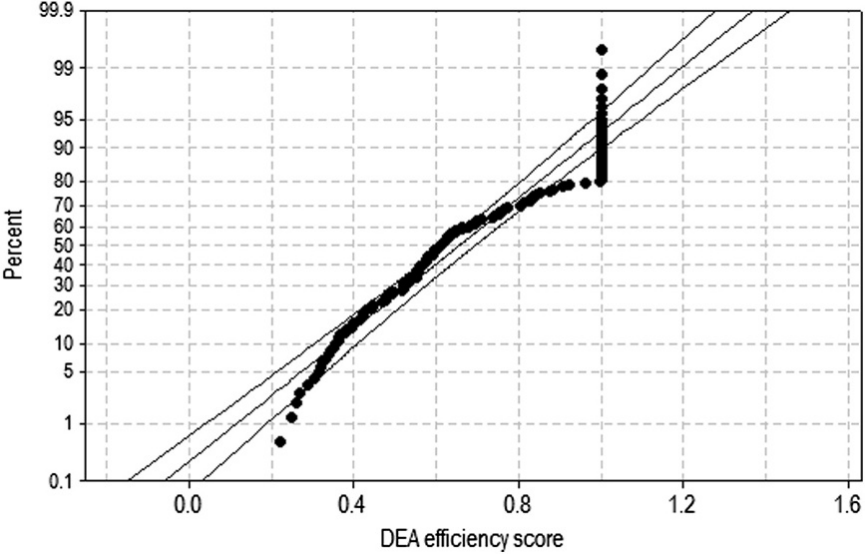
**Normal probability plot of DEA efficiency scores with the 95% CI (** ***n***_**1**_ **= 139).**

Again, in terms of DEA efficiency scores associated with the sample of *n*_1_ = 139, the Normality assumption should be rejected based on Figure 4 as well as two test statistics such as AD = 3.024^***^ (P-value < 0.005) and KS = 0.132^***^ (P-value < 0.010). Table 9 shows KW test results on DEA efficiency scores with the sample of *n*_1_ = 139 in Figure 4. Based on H = 27.04^***^ (P-value = 0.000), the efficiency difference between government subsidy sizes is statistically significant ().

**Table 9:** **Kruskal-Wallis tests on DEA efficiency scores (** ***n***_**1**_ **= 139)**

***i***	GS Class	***n***_***i***_	Median	Rank mean	Z _***i***_
1	GS-Class1	34	0.816	95.5	4.25^***^
2	GS-Class2	69	0.588	66.0	-1.15
3	GS-Class3	20	0.453	46.3	-2.84^***^
4	GS-Class4	8	0.794	85.3	1.10
5	GS-Class5	8	0.436	39.9	-2.18^**^
	Total	139		70.0	

H = 27.04^***^, DF = 4, P-value = 0.000 (tied-ranks adjusted).

For each level (i.e., each class of the government subsidy size), we can identify the individual level’s superiority against the others by calculating Eq.(6). As seen in Table 9,  of GS-Class1 exceeds the 99.5% percentile of the standard normal distribution, . Meanwhile, both  of GS-Class3 and  of GS-Class5 are less than the 2.5% percentile of the standard normal distribution, . In summary, even though there is an exception of GS-Class4, the phenomenon of diseconomies of scale is identified on the whole. More specifically, as the government subsidy size increases, the central measures of DEA efficiency scores are reduced, but the dispersion measures rather tend to get larger. Consequently, Hypothesis 2 in Section 2 can be strongly accepted with the statistical significance aforementioned.

**Figure 5:**
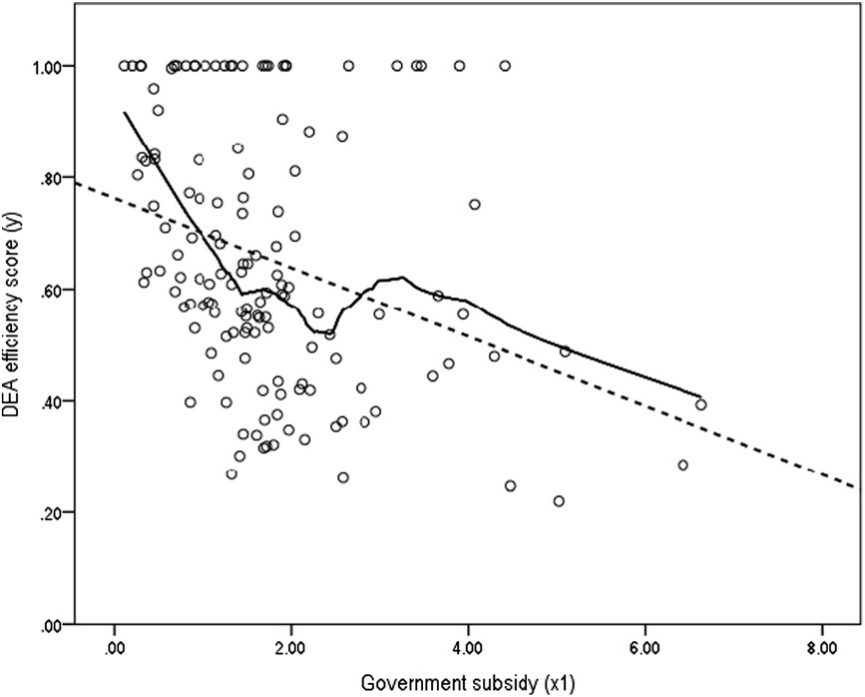
**Scatter plot of DEA efficiency score (** ***y*** **) versus Government subsidy (** ***x***_**1**_ **) (** ***n***_**1**_ **= 139).**

Figure 5 is a scatter plot of DEA efficiency score (*y*) versus Government subsidy (*x*_1_) with the sample of *n*_1_ = 139. In Figure 5, the dotted line represents the fitted regression equation, Eq.(7). Based on the standard error of ,  = 0.016 and its *t*-statistic = -3.863^***^, DEA efficiency score (*y*) is inversely proportional to Government subsidy (*x*_1_). Furthermore, applying the Epanechnikov weights using the sample proportion of 50%, we can add the locally weighted scatter plot smoother (LOWESS) irregular curve into Figure 5, which shows the same pattern as depicted in Figure 3 (IBM SPSS 2009).

7

Table 10 shows three different correlation coefficients of DEA efficiency score (*y*) versus Government subsidy (*x*_1_) with the sample of *n*_1_ = 139. Not only the parametric Pearson’s *r* = -0.313^***^ but also the other two nonparametric correlation coefficients including Spearman’s ρ_*s*_ = -0.369^***^ and Kendall’s τ_*B*_ = -0.264^***^ indicate a negative (-) correlation between the two series of values.

**Table 10:** **Correlation coefficients of DEA efficiency score (** ***y*** **) versus Government subsidy (** ***x***_**1**_ **) (** ***n***_**1**_ **= 139)**

Pearson’s *r*	-0.313^***^
(P-value)	(0.000)
Spearman’s ρ_*s*_	-0.369^***^
(P-value)	(0.000)
Kendall’s τ_*B*_	-0.264^***^
(P-value)	(0.000)

## Conclusions

The present study evaluated the efficiency of GSPs conducted by SMEs among KETIP 2012 of MKE in the Korean government. Among the total of 1,899 GSPs completed by SMEs in KETIP 2012, 139 GSPs were selected as the sample to be analyzed in the present study, which created three major performance factors (i.e., publications, patents and commercialization sales) during the past five performance follow-up survey years from 2007 to 2011. In particular, the present study aimed to answer the following question:“ Is there a difference in the efficiency between R&D collaboration types and between government R&D subsidy sizes?” Methodologically, a DEA model was established to measure the efficiency of each GSP, and DEA input and output variables were chosen by referring to typical program logic models and previous studies relating to national R&D programs. Next, for a major part of statistical analyses, KW tests were conducted to see if the efficiency differences were statistically significant between R&D collaboration types and between government R&D subsidy sizes. In addition, parametric and nonparametric correlation analyses were examined to verify the relationship between DEA efficiency scores and government subsidy sizes, which was captured by a fitted regression equation line as well as a LOWESS curve.

The major results of the analyses are as follows: First, two short-term and technical outputs such as publications and patents had a statistically significant positive (+) correlation with government subsidy, respectively, while the positive (+) correlation between commercialization sales and government subsidy was not statistical significant. Second, contrary to the first hypothesis, when the influence of government R&D subsidy size was controlled, there was no statistically significant difference in the efficiency between R&D collaboration types. However, JV-Type3 (i.e., SME-University-Laboratory Joint-Venture) was relatively superior to the others, which achieved the largest median as well as the smallest IQR of DEA efficiency scores. Third, the efficiency differences were statistically significant between government subsidy sizes, and the phenomenon of diseconomies of scale was identified on the whole. As the government subsidy size increases, the central measures of DEA efficiency scores are reduced, but the dispersion measures rather tend to get larger. In summary, Hypothesis 2 is accepted completely with the statistical significance. Meantime, Hypothesis 1 can be partially accepted based on the comparisons of the descriptive statistics of DEA efficiency scores. In particular, the nonparametric analysis framework proposed in this study and the findings by using this framework can be the major contribution of the present study to the literature.

There is a need to expand the analysis scope in the future other than SME. Additionally, future research could enhance the analysis by encompassing the efficiency comparison between groups of GSPs divided by the duration perspective (e.g., a group of short-term GSPs completed within two years versus the other group of long-term GSPs continuing over two years). In the meantime, a systematic search for influential factors other than the collaboration type and the government subsidy size that caused the efficiency difference was not included in the present study, which can be pointed out as one the limitations.

## Authors’ information

Sungmin Park received the Ph.D. degree in Industrial Engineering from Arizona State University (ASU), Tempe, Arizona, U.S.A.. He is currently an Assistant Professor at the Department of Business Administration at Baekseok University, Korea. Prior to his current position, he was a Senior Researcher at Samsung Electronics Co., Ltd.. Also, he was a Research Associate in the Modeling and Analysis of Semiconductor Manufacturing (MASM) Laboratory at ASU. He published in leading journals such as Operations Research, International Journal of Production Research, Simulation: Transactions of the Society for Modeling and Simulation International, IEEE Transactions on Semiconductor Manufacturing, International Journal of Simulation and Process Modelling, etc.. His research interests include operations management, especially for R&D performance evaluation.
